# In Memoriam—Dr. Samuel Mills Fowler

**Published:** 1899-07

**Authors:** 


### IN MEMORIAM—DR. SAMUEL MILLS FOWLER.

Dr. Fowler, a well known and distinguished veteran homœopathic physician, died at his residence, 619 East Sixty-sixth street, Chicago, Illinois, on Tuesday, March 28th, 1899, at six o'clock in the morning.

Dr. Fowler was an old soldier in the war of the rebellion, having entered the army as a private in Company F, Third Michigan Cavalry. In October, 1862, he was mustered out, but immediately re-enlisted and was finally discharged in San Antonio, Texas, in 1866. His attachment for his old comrades in arms continued all his life, and his work among these decrepit, prematurely-old, broken-down "boys" was untiring wherever he met them, and they in turn were equally devoted to him.

He was buried with their beautiful memorial service and they will erect a tablet to his memory in their magnificent memorial hall in the new Library at Washington. He wore a gold watch which was given him by these comrades, with an inscription upon the lid expressive of their esteem for him. This watch he gave to his first-born son on his twenty-first birthday, to be preserved by him as a family heirloom.

The war of the rebellion being over, Dr. Fowler began the study of medicine in the office of Dr. Sill in Kalamazoo, Michigan, and attended lectures in Cleveland Homœopathic Hospital College in 1870 and 1871. He graduated from the Hahnemann College in Chicago in 1872.

Whilst attending lectures at the Cleveland College he met his future wife, Miss Carrie H. Rogers, who was at the same college studying medicine. These two were married March 7th, 1872, in Dubuque, Iowa. Dr. Fowler was then 29 years old. He located first at Adrienne, Michigan. This proved unfortunate for his wife, who became a victim of ague. The young couple consequently removed to the home of the wife, Dubuque, Iowa, where Dr. Fowler went into partnership with Mrs. Fowler's preceptor, Dr. E. A. Guilbert.

In 1876 he dissolved this partnership and practiced with his wife. In 1883 Mrs. Fowler's mother was taken severely ill at DeLand, Volusia county, Florida. There Dr. and Mrs. Fowler repaired to the side of the invalid, at the same time practicing medicine and raising oranges.

Whilst living here an epidemic commonly called typho-malarial fever broke out in St. Augustine, and Dr. Fowler was tempted to go there to try the efficacy of Homœopathy. His wildest hopes were realized. Out of sixty-nine fever cases he lost but one, and that one had been abandoned by the allopathic attendant and lay for two days without any treatment. This experience made him very popular and very soon he had a fine practice. In 1888 yellow fever appeared and caused a panic among the people and they fled from the city, and his practice was soon reduced to very narrow limits.

Next he tried Gainesville, Texas, being persuaded to come by his brother and uncle. Here he had an excellent practice for two years, when a fire broke out in his dwelling that consumed all valuables, household goods and books, the accumulation of twenty years of married life. Whilst living in Gainesville he cured a number of cases given up to die by the old-school physicians. Among these was the infant child of the leading jeweler of the town. The child was kept in a stupor with Morphine injections to relieve spasms and had taken enough Gelsemium to scare the druggist who prepared it. The traditional mother-in-law was at hand and suggested the calling in of the "new doctor" and he gave the strictly homœopathic remedy and saved the babe.

But the conflagration having destroyed all he owned, Dr. Fowler no longer desired to live in a town where he had had such disaster.

Having heard of the formation of the new Hering Medical

College in Chicago and of Dr. Allen's desire for help in starting it, Dr. Fowler decided to move to Chicago. Nothing could be more in harmony with his enthusiasm in the cause of Homœopathy than to be one of the active spirits in conducting it and in having a chance to teach the doctrine in all its simplicity and accuracy, and incidentally to infuse into his hearers some portion of his own strength of conviction and loyalty to its principles. Therefore he was a most active and efficient teacher and inspiring spirit in the faculty. A disagreement having broken out in the faculty, a considerable number seceded and formed the Dunham Medical College. Dr. Fowler joined the new college and became professor.

In August, 1897, his rapidly failing health caused him to relinquish his professorship in the college and return to Florida, establishing himself at Miami. Here he continued to reside until December, 1898, when he came on a short visit to Philadelphia.

During his stay in this city he called upon the editor of The Homœopathic Physician. His appearance was shocking and eloquently told the story of his suffering. Notwithstanding his illness, he spent several pleasant evenings in our house and accepted our invitation to Christmas dinner. He heartily enjoyed this festival occasion and warmly expressed his pleasure. It was the last enjoyment he ever experienced, for the very next day his illness assumed so grave a phase that he telegraphed for his devoted and admirable wife to come to him. She responded promptly and on her arrival decided to take him away immediately to the home of his father, the Rev. Samuel Mills Fowler, Sr., in Kalamazoo, where he retired to his bed never to rise again. His sufferings were terrible and wrung the heart of his noble wife to the last extreme with anguish. Even though he is dead, the memory of those hours of pain have made a deeper impression and created a more poignant agony in this devoted and heroic woman than even the bereavement of his end and her own desolation.

After death an autopsy was held to determine the cause of his illness. The following is a copy of the certificate given by the surgeons who made the examination:

March 28th, 1899.

Examination of the body of Samuel Mills Fowler, M. D.

Spleen measured 8x6x4 inches; dense omental adhesions to the abdominal wall on the right side in the region of the cœcum. Appendix had degenerated into a fibrous cord. Liver greatly enlarged. A calculus 2½ inches long and 1½ inches in diameter completely filled the gall bladder, the walls of which invested it closely; the stone extended to and obstructed the common duct.

D. H. Galloway, Allop.,
W. H. Schrader, Allop.,
Howard Crutcher, Hom.,
Surgeons.

Dr. Fowler has the distinction of being one of the most learned men and one of the most enthusiastic homœopathists in the profession. He was one of the most accurate, painstaking, and therefore successful prescribers in our school.

He was the bitter foe of every innovation that was likely to lead the mind of the practitioner away from the strict detail of studying the case, comparing it with the materia medica, selecting the proper simillimum, and in the potentized dose, and watching the results of its careful administration. He had an abiding faith in the salutary results to be expected from the carefully selected remedy and in consequence he was able to save many a life for continued usefulness in this world, and also to stave off the time for the approach of the dread destroyer in his own case. His presence anywhere was the signal for the working of medical miracles in the relief of suffering and death. His meetings with his fellow-practitioners were enthusiastic conferences upon the powers of homœopathic principles, relations of his own successes in individual cases and the giving of valuable indications for remedies, or encouragement and good advice to his listener, if the latter were in doubt upon his own cases.

His widow has received from Dunham Medical College the following expression of feeling on the part of the faculty. Its brief sentences speak a volume of his character and attainments:

"CHICAGO, April 10th, 1899.

"MRS. S. MILLS FOWLER:

"DEAR MADAM—At the last faculty meeting of the Dunham Medical College the Registrar was requested to express to you the heartfelt sympathy and deep loss, not only our college, but the community sustained in the death of your good husband.

"Words at such a time can feebly express one's thoughts. His memory will ever be dear to hundreds, not only in Chicago, but everywhere. His sterling worth, splendid reputation and marked success in the practice of the Homœopathy of Hahnemann have left a record that few can hope to attain in this world.

"Yours very truly,

"JOHN STORER,

"Registrar."

